# Acute pulmonary histoplasmosis related to occupational roofing: A case report of two brothers

**DOI:** 10.18502/cmm.7.4.8409

**Published:** 2021-12

**Authors:** Martin Gnoni, Timothy McCann, Adrian Riva-Moscoso, Fortunato S. Príncipe-Meneses, Diego Chambergo-Michilot

**Affiliations:** 1 Department of Internal Medicine, Good Samaritan Hospital, Cincinnati, Ohio, United States of America; 2 Department of Medicine, University of Louisville Health Sciences Center, Louisville, Kentucky, United States of America; 3 Trihealth Internal Medicine, Infectious Diseases, Cincinnati, Ohio, United States of America; 4 School of Medicine, Peruvian University of Applied Sciences, Lima, Peru; 5 School of Medicine, Scientific University of the South, Lima, Peru

**Keywords:** Fungal, Histoplasmosis, Ohio, Spores, United States

## Abstract

**Background and Purpose:**

*Histoplasma capsulatum* is the cause of a prevalent fungal disease in certain regions in the United States of America, like Ohio and the Mississippi River. Its clinical manifestations range from asymptomatic to life-threatening diseases, according to the immune system. A definitive diagnosis is made by biopsy.

**Case report:**

Two middle-aged brothers presented with a nine-day history of severe progressive dyspnea. Both were living in Cincinnati, Ohio, and encountered bird droppings 7 days prior to symptoms while working on a roofing project. It should be mentioned that they were not wearing masks. After extensive testing, they were diagnosed with acute pulmonary histoplasmosis. Both were successfully treated with azole-derivative fungal therapy.

**Conclusion:**

This is the first case of histoplasmosis acquired through occupational exposure related to roofing and is unique given the two patients were siblings.

## Introduction

Histoplasmosis is a fungal disease caused by *Histoplasma capsulatum* [ 1 ]. It originates from exposure to spores from the feces of animals, like birds and bats. It can be classified as the environment- or occupation-related incidents [ 2 ]. Based on the reports of the Center for Disease Control and Prevention (CDC), up to 90% of people living in the Ohio and Mississippi River valley have been exposed to the infectious spores during their lifetime [ 3 ]. It is common for asymptomatic patients to present with granulomas found incidentally on chest x-ray or computed tomography [ 4 ]. However, diffuse acute pulmonary histoplasmosis can be a life-threatening disease [ 5 ].

Diagnosis is made by identification of the yeast using histopathology. Varying granulomatous responses can be seen on imaging. Serological and urine antigen tests are widely available; however, their sensitivity and specificity are poor [ 6 ]. To the best of our knowledge, this is the first case report of two simultaneous cases of occupationally acquired severe acute pulmonary histoplasmosis in two siblings of similar ages.

## Case report

Two brothers, a 47-year-old male (Case 1) with no significant past medical history, and a 45-year-old male (Case 2) with a past medical history of anxiety presented to TriHealth-Bethesda North hospital in Cincinnati, Ohio. Each had a nine-day history of progressive dyspnea. Their symptoms began with headaches and soon progressed to night sweats with fevers, fatigue, and exertional dyspnea. They encountered multiple bird nests with bird droppings while working on a roofing project. The symptoms appeared seven days after the exposure. It should be noted that they were not wearing masks. There was no history of recent travel to endemic regions or recent caving activities. They reported that they had not worked on any other jobs together. None of their co-workers or family members complained of similar symptoms.

### Case 1

**Figure 1:**
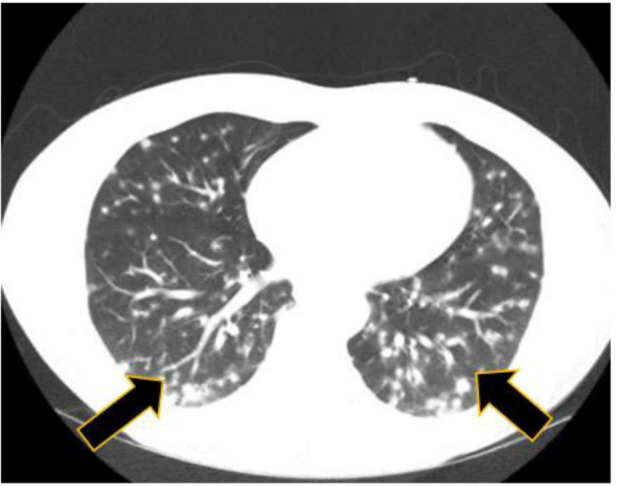
Patient 1 CT scan chest with evidence of diffuse granulomatous disease

**Figure 2:**
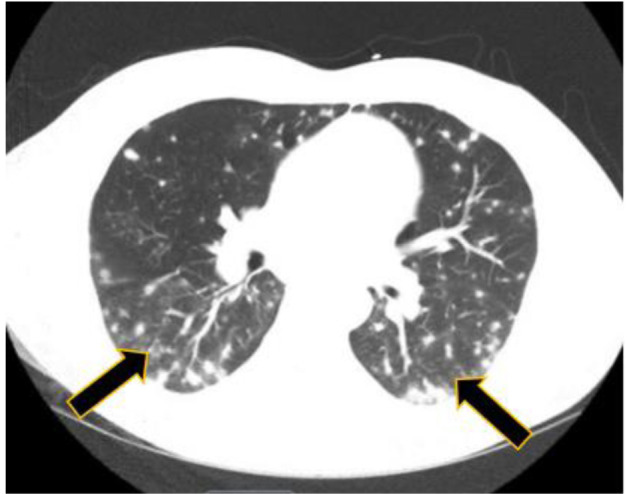
Patient 1 CT scan of upper chest with evidence of diffuse granulomatous disease

Initial laboratory evaluation showed a white blood cell count at 8,600 cells/µL with elevated absolute eosinophils at 0.17 cells/µL. Elevated transaminases were present with an alanine aminotransferase of 197 IU/L and aspartate transaminase of 84 IU/L. Computed tomography evaluation of the thoracic region showed extensive miliary-type pulmonary nodules with ground-glass opacities (figures 1 and 2). Laboratory evaluation on day 2 included a negative *Histoplasma* serum antigen and a positive *Blastomyces* serology. Urine *Histoplasma* antigen was never performed due to a laboratory error. A bronchoalveolar lavage (BAL) performed on day 3 of hospitalization yielded a negative direct microscopic examination, followed by growth of *H. capsulatum* at 14 days of culture (Table 1).

**Table 1:** Variation in testing positivity between patients

Test	Patient 1	Patient 2
Bronchoalveolar lavage (BAL) culture	*Histoplasma growth*	Negative
Urine *histoplasma* antigen	Not sent	Negative
Serum *histoplasma* antigen	Negative	Positive
Serum *blastomyces* Serology	Positive	Positive

### Case 2

**Figure 3:**
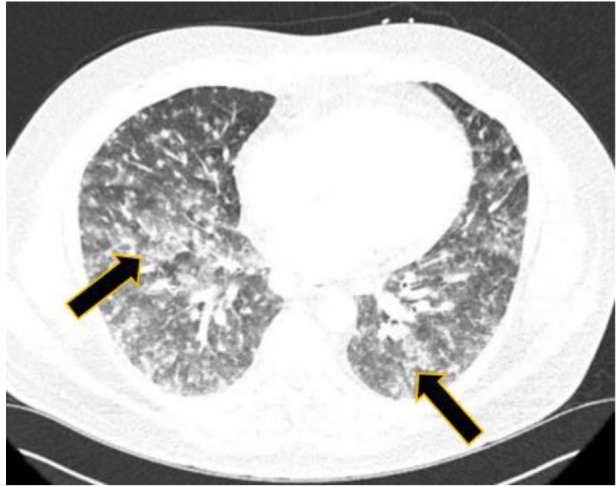
Patient 2 CT scan of chest with evidence of granulomatous disease

**Figure 4:**
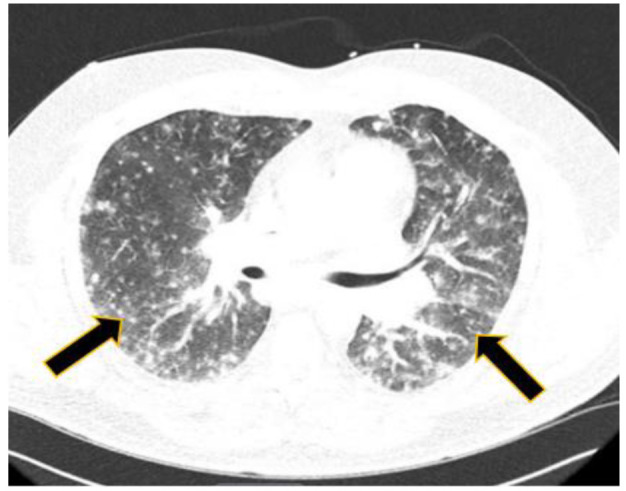
Patient 2 CT scan of chest with evidence of granulomatous disease

Initial laboratory evaluation showed leukocytosis at 14,300 cells/µL with an elevated absolute eosinophil count of 0.17 cells/µL. Elevated transaminases were present with alanine aminotransferase of 165 IU/L and aspartate transaminase of 54 IU/L. There were mildly elevated acute phase reactants with an erythrocyte sedimentation rate at 22 and C-reactive protein at 30. The computed tomography evaluation of the thoracic region revealed extensive miliary-type pulmonary nodules and accompanying hilar and subcarinal adenopathy (figures 3 and 4).

Positive tests included *Histoplasma* serum antigen of 5.88 ng/ml (range: 0.19-60.0 ng/ml) and positive *Blastomyces* serology performed on day 2 of hospitalization. Urine *Histoplasma* antigen test was negative (day 2). A direct microscopic examination of BAL fluid performed on day 3 of hospitalization as well as cultures (at 4 weeks) yielded negative results (Table 1).

Both cases started on treatment with broad-spectrum antifungal therapy on day 1 of hospitalization with liposomal amphotericin B (3-5mg/kg/day) for 5 days and were quickly transitioned to oral itraconazole 200 mg twice daily. Lengths of hospital stays were less than 7 days; however, patient 2 returned to the hospital due to medication non-compliance 7 days after discharge due to concerns of medication interaction with itraconazole and alprazolam. He was then started on isavuconazole 200 mg daily. 

Both patients had excellent clinical responses to 12 weeks of oral azole therapy. Both of them had negative follow-up *Histoplasma* antigen testing 3 months after therapy. They remain asymptomatic and are currently being monitored off antifungal therapy. It should be mentioned that written informed consent for publication was obtained from both patients.

## Discussion

Some endemic fungal infections (especially *H. capsulatum* and *Blastomyces dermatitidis*) tests cross-react. It is common for clinicians to diagnose either condition based on prior knowledge of this cross-reactivity. This is primarily due to the similarity of certain structures of the cell wall of these organisms. Results of a study showed that cross-reactions with histoplasmosis occurred in 90% of patients with proven pulmonary and/or extrapulmonary blastomycosis [ 7 ]. Additionally, 80%, 60%, and 10% of patients have cross-reactivity with *Paracoccidioides*, *Coccidioides*, and *Aspergillus*, respectively [ 7 ]. 

The gold standard diagnosis of histoplasmosis is identification by culture. However, it must be noted that culture sensitivity remains low [ 8 ]. This is noted in the discrepancies seen in both case cultures. Urinary and plasma *Histoplasma* antigens are easy and cost-effective ways to achieve a diagnosis. It is known that the urinary antigen has higher sensitivity than the plasma antigen [ 9 ]. In a meta-analysis, it was found that antigenuria and antigenemia have high specificity (98.8% and 97.5%, respectively). The remaining cases can present as false positives (1.7%) due to possible cross-reactivity with *Paracoccidioides* or *Blastomyces* [ 9 ]. One strategy is to run a Histoplasma antigen detection in the bronchoscopy fluid, which has a sensitivity of 93.5% [ 10 ]. 

In case 1, we expected a positive urinary antigen due to the high burden of disease with positive cultures, but it was not sent. The serum antigen was negative, potentially because the concentration did not reach the threshold of positivity (usually it is less sensitive than the urinary antigen). In case 2, the serum antigen was positive; however, the BAL cultures were negative. The variance in these results may be related to a multitude of factors, including the time of collection of samples related to treatment initiation, type of sample, the burden of disease, intrinsic characteristics of the test, or immune response of the host to the infection. 

Itraconazole increases the half-life of alprazolam. In one study, it was found that the half-life of alprazolam was 40.3 h in patients who received itraconazole, compared to 15.7 h of those patients who received placebo. Itraconazole has been demonstrated to inhibit CYP3A (a mechanism responsible for the primary metabolism of alprazolam) [ 11 ]. This interaction represents a risk for alprazolam toxicity, risking central nervous system depression. Due to the drug-drug interaction, we favored the use of isavuconazole. Even though the clinical and antigenemic response was favorable, itraconazole remains the first choice for this disease according to the guidelines of the Infectious Diseases Society of America [ 12 ]. Alternative options should be pursued only in severe drug-drug interaction cases, intolerance, or adverse drug effects [ 12 ]. Here, the interaction class was “severe” between alprazolam-itraconazole and “moderate” between isavuconazole-alprazolam [ 13 ].

Case reports of occupational histoplasmosis have been previously reported [ 14 ]. In the Ohio river valley, an outbreak was reported among bridge builders in 2003, and close contact with bat guano was thought to be the cause [ 15 ]. In the Dominican Republic, tunnel workers developed histoplasmosis within days of exposure to bat guano. In 2004, an outbreak was reported at an agricultural plant. It was noted that only 25 out of 978 workers developed acute histoplasmoses based on confirmatory laboratory tests. 

Acute histoplasmosis can develop from high inoculum inhalation in immunocompetent hosts [ 15 - 16 ]. In the USA, the incidence rate is 6.1 cases per 100,000 [ 6 ]. The U.S. National Institute for Occupational Safety and Health has established measures to prevent occupational histoplasmosis among workers, including, but not limited to the use of N95 masks. The CDC guidelines on the prevention of histoplasmosis have divided the risk into 3 categories: high, low, and minimal/no risk. The use of personal protective equipment varies between the categories. The recommendations range from disposable coveralls, rubber boots, and high-efficiency particulate air respirators to the ability of not using mandatory personal protective equipment [ 17 ]. 

## Conclusion

This is the first documented case of occupation-related acute pulmonary histoplasmosis related to roofing. It is further unique in the fact that the patients were not only co-workers but siblings of similar age. The authors highlight the exposure risk of certain professions as well as the complexity of the diagnosis and treatment of endemic fungal infections. We aim to emphasize the limitation of the current tests available for the diagnosis of histoplasmosis as well as the treatment difficulties associated with using itraconazole, specifically its drug-drug interactions. Through patient advocacy and support, we aim to raise awareness of the occupational hazards of roofing and acquire histoplasmosis so that proper personal protective equipment can be utilized within at-risk populations.

## Acknowledgement

None.

## Authors’ contribution

G. M. and M. T contributed to study conception and design. R. M. A. and P.M. F. S. and C. M. D. designed the outline and coordinated the writing of the paper. All authors wrote the original manuscript and assisted in editing. M.T. prepared the figures. G. M. and M.T. supervised the majority of the writing and provided critical reviews.

## Conflict of Interest

The authors declare no conflicts of interest.

## Financial disclosure

This research received no specific grant from any funding agency in the public, commercial, or not-for-profit sectors.

## Ethical Considerations

Written informed consent was obtained for both patients. 
